# Displacing the Burden: A Review of Protein-Bound Uremic Toxin Clearance Strategies in Chronic Kidney Disease

**DOI:** 10.3390/jcm13051428

**Published:** 2024-03-01

**Authors:** Didier Sánchez-Ospina, Sebastián Mas-Fontao, Carolina Gracia-Iguacel, Alejandro Avello, Marina González de Rivera, Maddalen Mujika-Marticorena, Emilio Gonzalez-Parra

**Affiliations:** 1 Servicio Análisis Clínicos, Hospital Universitario de Burgos, 09006 Burgos, Spain; sliverco41@gmail.com (D.S.-O.); mmujika@saludcastillayleon.es (M.M.-M.); 2 IIS-Fundación Jiménez Díaz, 28040 Madrid, Spain; smas@fjd.es; 3 Centro de Investigación Biomédica en Red de Diabetes y Enfermedades Metabólicas Asociadas (CIBERDEM), 28029 Madrid, Spain; 4 Faculty of Medicine and Biomedicine, Universidad Alfonso X el Sabio (UAX), 28037 Madrid, Spain; 5 Department of Nephrology and Hypertension, IIS-Fundación Jiménez Díaz, Univerdad Autonoma de madrid, 28049 Madrid, Spain; cgraciai@quironsalud.es (C.G.-I.); alejandro.avello@quironsalud.es (A.A.); marina.grivera@fjd.es (M.G.d.R.)

**Keywords:** chronic kidney disease, uremic toxins, protein-bound uremic toxins, adsorptive therapies, molecular displacers

## Abstract

Uremic toxins (UTs), particularly protein-bound uremic toxins (PBUTs), accumulate in chronic kidney disease (CKD) patients, causing significant health complications like uremic syndrome, cardiovascular disease, and immune dysfunction. The binding of PBUTs to plasma proteins such as albumin presents a formidable challenge for clearance, as conventional dialysis is often insufficient. With advancements in the classification and understanding of UTs, spearheaded by the European Uremic Toxins (EUTox) working group, over 120 molecules have been identified, prompting the development of alternative therapeutic strategies. Innovations such as online hemodiafiltration aim to enhance the removal process, while novel adsorptive therapies offer a means to address the high affinity of PBUTs to plasma proteins. Furthermore, the exploration of molecular displacers, designed to increase the free fraction of PBUTs, represents a cutting-edge approach to facilitate their dialytic clearance. Despite these advancements, the clinical application of displacers requires more research to confirm their efficacy and safety. The pursuit of such innovative treatments is crucial for improving the management of uremic toxicity and the overall prognosis of CKD patients, emphasizing the need for ongoing research and clinical trials.

## 1. Introduction

Chronic kidney disease (CKD), a global health challenge, impacts an estimated 10–15% of the world’s population [1]. In 2017, around 843.6 million individuals were affected, as recent studies on the global CKD prevalence indicate [2]. This rise is partly attributed to the increased incidence of diabetes, hypertension, obesity, and aging populations [3], alongside improved access to renal replacement therapies in economically developing nations [4].

CKD stages are independently linked to heightened cardiovascular event risks, decreased quality-adjusted life years, and high morbidity and mortality rates. From 1990 to 2017, the CKD-related global mortality surged by 41.5%, ranking it as the 12th leading death cause worldwide [5]. By 2040, it is projected to become the 5th leading cause of global mortality [6].

One CKD consequence is the gradual decline of glomerular filtration, leading to metabolic waste product accumulation in the bloodstream, known as uremic toxins (UTs). These toxins are associated with the uremic syndrome, presenting symptoms like nausea, vomiting, asthenia, anorexia, and pruritus due to their detrimental pathophysiological effects [7].

## 2. Definition and Classification of Uremic Toxins

Definition of Uremic Toxins: Uremic toxins (UTs), pivotal to the pathology of chronic kidney disease (CKD), are metabolic by-products typically excreted by healthy kidneys. In CKD, however, diminished glomerular filtration, particularly at rates below 60 mL/min/1.73 m^2^, results in their accrual in the bloodstream. UTs emerge from diverse origins, including the degradation of endogenous and bacterial proteins and the consumption of certain foods [8]. The complex pathophysiological mechanisms they trigger include inflammation, oxidative stress, cellular trans-differentiation, mitochondrial dysfunction, intestinal barrier impairment, and gut microbiota alterations [9,10].

In 1999, the European Uremic Toxins (EUTox) working group, spearheaded by Vanholder and colleagues, established a classical definition and classification of UTs. This definition, recently scrutinized for its breadth and precision, laid out five criteria for classifying an organic solute as a uremic toxin:

(a) Chemical Identification and Analysis: The compound must be chemically identifiable, with quantitative analysis feasible in biological fluids.
(b) Elevated Levels in Uremia: The total and plasma levels should be higher in uremic subjects than in non-uremic individuals.
(c) Clinical Relevance: Elevated concentrations should correlate with specific uremic dysfunctions and/or symptoms that decrease or disappear when the concentration is reduced.
(d) Biological Activity: There must be evidence of biological activity, consistent with clinical changes observed in uremic syndrome, demonstrated in in vivo, ex vivo, or in vitro studies.
(e) Concentration Consistency: Concentrations in these studies should reflect those found in bodily fluids or tissues of uremic patients.

In 2003, EUTox introduced a UT classification based on the physicochemical properties influencing their clearance during conventional hemodialysis [11]:

- Small Hydrophilic Toxins (<500 Da): These include compounds like urea (60 Da) and uric acid. Conventional hemodialysis effectively removes them using diffusion as the primary transport mechanism [12].
- Medium-Sized Toxins (≥500 Da): Examples are β2 microglobulin (11.8 kDa) and parathyroid hormone (9.5 kDa). While convective transport can remove some of these toxins, their size hinders efficient elimination [13].
- Protein-Bound Toxins (PBUTs): This category encompasses molecules with low molecular weight, such as indoxyl sulfate and p-cresyl sulfate, which exhibit more than 80% plasma protein binding. Despite their inherently low molecular weight, clearance is negatively affected due to the lower concentration of unbound toxin at the dialysate side surface of the membrane.

The expanding knowledge of UTs, with over 120 molecules identified to date [14], coupled with advancements in hemodialysis techniques, has necessitated a re-evaluation of the definition and classification of uremic retention solutes [15]. The prior classification’s limitations, such as the inaccuracy in capturing the variable protein binding of uremic solutes and its application solely to conventional hemodialysis, are now being addressed. This re-examination considers factors like solute compartmentalization within the body and alternative strategies for uremia reduction, such as preserving residual renal function and employing adsorption and convection techniques. Some of the most relevant PBUTs for dialysis adequacy are shown in Table 1.

## 3. Protein-Bound Toxins: Main Types and Molecular Weight

Characteristics of Protein-Bound Uremic Toxins (PBUTs): Protein-bound uremic toxins (PBUTs) are a distinct class of toxins characterized by their strong affinity to plasma proteins, particularly albumin. This binding complicates their elimination via conventional dialysis techniques. The European Group for the study of Uremic Toxins (EUTox) recognized 25 PBUTs in 2003 [11], further categorizing them based on their originating compounds into groups like phenols, indoles, hippurates, polyamines, advanced glycation end products (AGEs), and peptides or small and medium-sized proteins, including leptin and retinol-binding protein. The binding affinity of these toxins for albumin varies, with indoxyl sulfate (IS), 3-carboxy-4-methyl-5-propyl-2-furanpropanoic acid (CMPF), p-cresyl sulfate, hippuric acid, and indoleacetic acid exhibiting the highest affinity.

Formation and Elimination: The genesis of PBUTs primarily occurs in the intestine, where dietary proteins undergo metabolism by the intestinal microbiota, producing precursors that later form toxins. This intestinal origin underscores the increasing importance of studying the gut microbiome in preventing renal disease [17]. The plasma levels of PBUTs consist of the free fraction and the protein-bound fraction, with the toxicity largely attributed to the free fraction. This aspect becomes particularly significant in malnourished patients with hypoalbuminemia, as they exhibit a higher concentration of the free fraction, leading to more severe uremic symptoms.

Molecular Weight and Protein-Binding Considerations: PBUTs typically have a molecular weight under 500 Da. However, their protein binding confers them with an effectively larger molecular size. Notably, the peptide group among PBUTs, including leptin and retinol-binding protein, exhibits significantly higher molecular weights of 16,000 Da and 21,200 Da, respectively.

**Figure 1:**
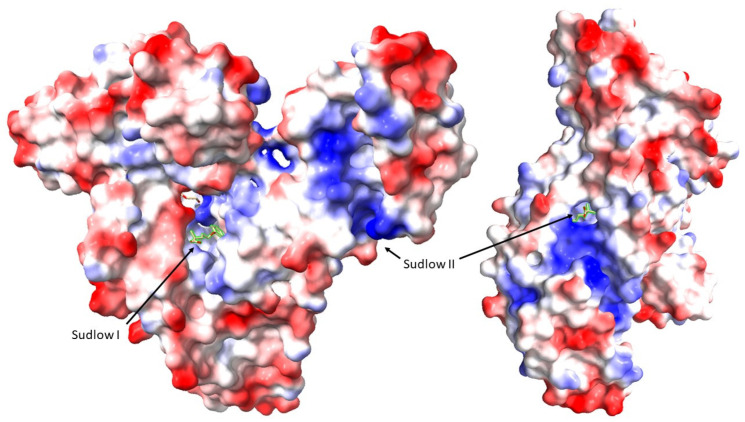
Location of Sudlow sites I and II (black arrows) on a human albumin molecule complexed with Gemfibrozil (PDB ID: 7QFE) [27], depicted using Coulombic electrostatic potential (ESP) color coding (coloring gradient that spans from red, indicating negative potential to blue, signifying positive potential) in ChimeraX.

Being the most abundant plasma protein, albumin plays a crucial role in binding various compounds, including uremic toxins and drugs, due to its two binding sites for toxins: one high-affinity site and one low-affinity site and specificities [18]. It has multiple binding sites that can interact with uremic toxins, including at least two drug-binding sites [19]. The most studied binding sites, known as site I and site II, have been identified for their ability to bind a variety of drugs and metabolites [20]. Additionally, modeling suggests that albumin contains two binding sites for toxins, a single high-affinity site and a second low-affinity site [21] (Figure 1).

Affinity and Specificity: Interactions between albumin and toxins can be hydrophobic, electrostatic, or through hydrogen bonding. Studies have also shown that albumin has specific binding sites for anionic, neutral, and cationic ligands, indicating its versatility in binding different types of compounds [22]. The nature and strength of these interactions depend on the chemical structure of the toxin and the physiological conditions, such as the pH and the presence of other ligands. Several factors can influence albumin’s ability to bind uremic toxins:

(a) Post-Translational Modifications: Glycation, oxidation, and other changes in albumin can alter its structure and, therefore, its binding capacity. In patients with chronic kidney disease, these modifications are more common and can affect albumin’s transport function [20].
(b) Competition with Other Molecules: The presence of drugs and other metabolites in plasma may compete with uremic toxins for binding sites on albumin, affecting its ability to neutralize these toxins [23,24].
(c) Changes in pH and Electrolytes: Variations in the blood pH and electrolyte levels, common in patients with renal insufficiency, can modify albumin’s structure and its affinity for uremic toxins. Albumin’s spatial structures are sensitive to changes in the acid–base balance, common in patients with renal insufficiency, and their tertiary structures change considerably with pH variations [25,26].

## 4. Effects of Protein-Bound Uremic Toxins

Protein-bound uremic toxins (PBUTs) are not merely waste products; their accumulation in chronic kidney disease (CKD) can lead to a range of systemic effects. These toxins, particularly notorious for their harmful influence on various tissues, significantly impact the cardiovascular system. Studies have elucidated the multifaceted roles of PBUTs in instigating renal fibrosis, vascular calcification, anemia, peripheral arterial disease, adynamic bone disease, insulin resistance, malnutrition, and immune system deficiency [7,28].

- Endothelial Dysfunction: Endothelial dysfunction caused by PBUTs is closely related to the development of cardiovascular diseases in patients with chronic kidney disease. PBUTs can cause structural damage, inflammation, and a decrease in endothelium-dependent vasodilation [29,30]. Furthermore, endothelial dysfunction is associated with the progression of chronic kidney disease and albuminuria [31]. Patients undergoing dialysis for chronic kidney disease exhibit a markedly diminished endothelial response to stimuli when compared to a control group of healthy individuals. This reduced response is evident across various assessment parameters, including both shear stress and biochemical agents, indicative of compromised endothelial function [32]. PBUTs can decrease nitric oxide production in endothelial cells by inhibiting endothelial nitric oxide synthase (eNOS) activity and expression [33]. PBUTs, like indoxyl sulfate, act as prooxidant and proinflammatory agents, which are associated with changes in the hemostatic system, increased oxidative stress, and monocyte activation. Additionally, this leads to a prothrombotic state through the activation of prothrombotic factors such as tissue factor and factor Xa [34], and the formation of endothelial microparticles.

High levels of indoxyl sulfate (IS) and p-cresol sulfate (PCS) in the serum have been used to predict cardiovascular events and are also implicated in vascular disease, including arteriosclerosis, endothelial inflammation, oxidative stress, and vascular calcification [35].

Most cardiovascular complications associated with chronic kidney disease are secondary to the activation of prooxidative/inflammatory pathways through human AhR activation. PBUTs have been recognized as endogenous agonists of AhR [36]. The aryl hydrocarbon receptor (AhR) is a transcription factor found in the cell’s cytoplasm in its inactive form. It has been demonstrated that AhR is more stimulated in stage 3 chronic kidney disease patients, directly associated with higher IS levels and inversely proportional to epidermal growth factor receptor (EGFR) levels [37].

- Prooxidant and Proinflammatory Actions: Indoxyl sulfate acts as both a prooxidant and proinflammatory agent, linked with changes in the hemostatic system, increased oxidative stress, and monocyte activation. This leads to a prothrombotic state through the activation of prothrombotic factors such as tissue factor and factor Xa [34], and the formation of endothelial microparticles.
- Cardiorenal Syndrome: The accumulation of PBUTs, particularly IS, in cardiomyocytes is linked to increased production of inflammatory cytokines such as IL1, IL6, and TNF-α [38]. These toxins have been associated with pro-arrhythmogenic effects and atrial fibrillation [35]. Studies have also noted structural and functional changes in cardiomyocytes, including reduced spontaneous contraction and irregularity, following exposure to toxins like p-cresol sulfate (PCS) [39].
- Immune System Dysfunction: Patients with chronic kidney disease present immune system dysfunction due to various causes, such as the dialysis process, vitamin D deficiency, and a sustained systemic inflammatory state due to elevated PBUT, which can alter the innate immune response [40]. Among the main PBUTs related to immune system activation are IS, PCS, and p-cresyl glucuronide, among the most well-known [41,42].

IS acts as a prooxidant and proinflammatory agent, triggering immune responses and stimulating chronic kidney disease progression. Increased plasma IS has been associated with changes in the coagulation cascade, increased oxidative stress, and monocyte activation [43]. This molecule shows a positive correlation with neopterin, a molecule generated by macrophages and monocytes after being stimulated by IFN-gamma produced by activated T cells. As a result, a high production of reactive oxygen species (ROS) and an increase in the expression of cell adhesion molecules (CAM) can be observed, promoting monocyte–endothelial cell interaction, leading to vascular inflammation and endothelial dysfunction. PBUTs affect both the innate and adaptive immune systems through multiple mechanisms, resulting in the development of systemic pathologies in humans, highlighting the importance of studying them, and advancements in this field would greatly improve the clinical management of these patients.

### Kidney

Accumulation of indoxyl sulfate (IS) can lead to the deterioration of the remaining renal nephrons, primarily within proximal tubular cells, thereby stimulating glomerulosclerosis, renal fibrosis, and the progression of chronic kidney disease (CKD). This process contributes to an increased expression of pro-collagen alpha 1, transforming growth factor beta 1 (TGF-β1), and tissue inhibitor of metalloproteinase 1 (TIMP-1) genes, resulting in further nephron loss and thereby accelerating CKD progression [44]

There is evidence that elevated levels of p-cresyl sulfate (PCS) in the kidneys lead to increased expression of proinflammatory cytokines and genes in renal tubular cells, along with activation of the renin–angiotensin–aldosterone system (RAAS) and epithelial–mesenchymal transition, culminating in fibrosis and nephrosclerosis [45]. Moreover, elevated PCS levels are associated with reduced Klotho expression through methylation of the Klotho gene, contributing to renal cell senescence [46].

## 5. PBUT Clearance Strategies

Conventional dialysis remains the primary treatment modality for patients with end-stage chronic kidney disease (CKD). Nevertheless, the effective removal of protein-bound uremic toxins (PBUTs) presents a significant challenge in these patients, attributed to their high affinity for protein binding. This limitation is not adequately addressed by current conventional methods. There is a scarcity of long-term evidence, as most efforts to enhance the clearance of these toxins remain experimental. Furthermore, regarding the techniques currently employed, such as prolonged and frequent dialysis, there are no comprehensive studies that evaluate the long-term outcomes of PBUT removal in comparison to other techniques [47].

### 5.1. Conventional Dialysis Efficacy on Protein-Bound Uremic Toxins (PBUTs)

Conventional dialysis methods, including hemodialysis and peritoneal dialysis, have not been proven effective in significantly reducing the levels of PBUTs [48,49]. When focusing on protein-bound toxins, it becomes evident that they play a critical role in patients undergoing dialysis due to the inability of the dialysis membrane to filter them effectively. Various studies have focused on this issue, adopting different approaches [50]. Although advancements in membrane technology and purification techniques have shown varying degrees of success in decreasing the free fraction of certain toxins, the clinical significance of these reductions remains under active investigation. Some membranes, especially those with high sieving coefficients, have been promising in enhancing toxin purification. However, their overall efficacy varies depending on the toxin type and clinical scenario, see Table 2 [51].

It appears that no membrane or technique, regardless of its high sieving coefficient, has been able to adequately purify these toxins. They may demonstrate a reduction in the free fraction, but this represents a very modest clinical impact, as the free fraction constitutes a minimal portion of the total toxin amount. However, the use of albumin in dialysate, by promoting binding with a high flow, demonstrates that standard dialysis membranes are not the limiting factor due to the low molecular weights of PBUTs but rather its protein binding [51].

In recent years, the clearance profiles of state-of-the-art hemodialysis membranes have seen significant improvements. Several characteristics must be considered in the evaluation of new membranes. These include new permeability rates, the hydrophilic or hydrophobic nature of the membranes, adsorption capacity, and electrical potential [65]. Additionally, the onset of molecular weight retention, molecular weight limit, and mass transfer area coefficient must be measured [66].

Conventional dialysis poorly clears them because only the free solute portion contributes to the concentration gradient that drives their diffusion from plasma to dialysate. The extent to which protein binding limits the removal of PBUTs depends on multiple factors, including the dialyzer size, dialysate flow, and the strength of the protein binding itself. Despite the rapid dissociation of PBUTs from albumin, studies by T. Meyer demonstrate that significantly increasing the dialysate flow with standard dialyzers can approximately quadruple the PBUT removal [51].

### 5.2. Conventional Dialysis: Importance of Dialysis Time

One of the most crucial factors in the efficacy of uremic toxin elimination is the dialysis time. The duration of the dialysis session is a critical determinant to ensure adequate clearance. Generally, longer dialysis sessions allow for more effective removal of PBUTs [67]. The reason is that the small-sized free fraction is cleared, balancing with the albumin-bound fraction released from anchoring to maintain the free fraction ratio. The longer the hemodialysis (HD) session, the progressively more free fraction is cleared. The clearance is the same per minute but is more constant and frequent [47]. Dissociation of the protein-bound form requires time; a conventional dialysis session is too short to prevent the new equilibrium of PBUTs strongly bound to albumin [68]. But to drive this transfer, what is needed is merely more clearance of the free fraction of the PBUTs within the dialyzer (as with more frequent dialysis with the same blood, more time on dialysis, or increasing the dialysate flow). PBUTs’ clearance increases when the free form is removed in long conventional dialysis, while it does not change with extended convective dialysis [52]. Association/dissociation of PBUTs and albumin happens during the time of blood passing through a hollow fiber dialyzer, when a strong chemical gradient is promoted by a rapid dialysate flow.

Prolonging the HD time through extended nocturnal HD removes a larger amount of PBUTs. Cornelis et al. observed higher PCS and IS clearance in long nocturnal dialysis, although the plasma concentrations did not change when the HD duration increased from 4 to 8 h [69]. Much of the problem is also in the slow diffusive transfer of PBUTs and mid-large dialysate toxins from cells to interstitium to blood. Long dialysis also provides time for this transfer [70].

### 5.3. Importance of Residual Renal Function

Residual renal function is important in reducing PBUTs [53,54]. The native kidney eliminates PBUTs mainly as free forms, while the total forms of IS and pCS are eliminated only 2% and 1.7%, respectively. Dialysis clears the total forms similarly to the native kidney, while it clears only 20–30% of free forms compared to the native kidney [55].

### 5.4. Online Hemodiafiltration: Role of Convection

Convection increases the elimination of uremic toxins during dialysis, especially medium- or large-sized ones [71]. However, PBUTs’ clearance with convective techniques has not shown conclusive data on their efficacy. One study demonstrated a lower pCS concentration and higher elimination in predilutional 60 L online hemodiafiltration compared to postdilutional 20 L. In addition to free PBUTs, small-sized toxins, including urea and creatinine, are better eliminated in predilutional HDF than postdilutional [52]. However, another study showed a greater reduction in both free and protein-bound PBUTs in postdilutional online HDF [56].

### 5.5. Expanded HD

Medium cutoff (MCO) dialyzers, also known as expanded dialysis, cannot increase PBUT elimination [57,58].

### 5.6. Adsorptive Therapies

Adsorptive therapies represent an innovative strategy for addressing uremic toxin removal in CKD patients. Despite their effectiveness, technical complications such as cost, biocompatibility and material saturation limit their use (Table 2).

These therapies rely on the ability of certain adsorbent materials to selectively capture PBUTs from the bloodstream, not only the free fraction but also the protein-bound fraction, due to their high affinity for these molecules [72].

The mechanism of action involves the interaction between PBUTs and adsorbent materials. When the patient’s blood or dialysate flows through an adsorptive therapy device, uremic toxins bind to the adsorbent surface due to chemical and physical forces. Once bound, they do not detach, causing material saturation depending on the surface. Activated carbon’s high adsorption capacity and other adsorbent materials have led to a significant reduction in toxin concentrations in CKD patients [73,74,75,76].

Among the adsorbent materials used is activated charcoal, which significantly improves toxin clearance when used simultaneously with conventional HD [61] or with hemo-perfusion [62]. Activated carbon has a high specific surface area and exceptional adsorptive properties [77].

Besides charcoal, many molecules, primarily celluloses or polymers, have been used. Hexadecyl chains immobilized in cellulose pores have been used simultaneously with conventional HD, resulting in a 34% decrease in the free form of IS, while the total IS barely changed [62].

CMK-3 is a silica- and carbon-based nanoporous sorbent [78]. The CMK-3 sorbent presents two different types of pores, micropores and mesopores [63], showing a high adsorption level on the free fraction of PBUTs. In another study [64] with two different resins, one with a sorbent based on divinylbenzene attached to a highly biocompatible polymer and cellulose with hexadecyl chains, showed a significant reduction in the free form rather than the total PBUTs. The difficulty in reducing the total PBUTs could be due to the constant disturbance of the balance between the free and protein-bound forms [68]. Initially, the unbound fraction undergoes elimination, resulting in a disruption of equilibrium between the bound fraction and the extravascular compartments. The dissociation does not occur until the concentration of the unbound fraction decreases, a process that unfolds gradually due to its dialysis over the course of the session. Despite the rapid dissociation capacity of albumin [79], the equilibrium is eventually restored as the bound fraction is gradually released. However, the passage of toxins from the tissue compartment to the blood is very slow and constitutes the most limiting factor. The degree of binding is related to the concentration of PBUTs around the albumin. As the unbound concentration decreases, especially below the dissociation constant level, the PBUTs have to leave the albumin. If a sorbent treatment removes free PBUTs but not the total, it is because the albumin has bound PBUTs in its course around the body. This mechanism may elucidate the augmentation in the binding percentage observed in certain studies, along with the potential modulation of equilibrium by variables such as the pH [68].

Efforts have also been directed toward enhancing this adsorption process through the application of prior plasma separation. While this method has shown promise, it is characterized by its labor-intensive and costly nature [75,76]. T. Meyer has observed that using a conventional high-permeability dialyzer and standard dialysis system provided total solute clearances of about 18 mL/min for p-cresol sulfate, and 19 mL/min for indoxyl sulfate, when dialyzing blood with these tightly bound solutes [51]. The dialyzers with a carbon-block recirculating system had clearances of about 45 mL/min for p-cresol sulfate and 61 mL/min for indoxyl sulfate when operating alone, without removing small toxins such as urea. When operated in series, the clearances of the carbon-regenerated dialysis system and regular dialysis system had clearances for PBUTs that were additive. These clearances were with standard high-permeability dialyzers, and the only change was the increase in dialysate flow rate to 1000 mL/min that is made possible by regeneration of the dialysate by an activated carbon block. So, 80% binding or even 90% is not so high that significant clearances are made impossible with standard dialysis membranes. A high dialysate flow rate maintains the gradient for removal by diminishing the dialysate concentration right at the membrane surface. Suspended charcoal particles in the dialysate can do the same thing as a very high dialysate flow rate [51]

#### Challenges and Future Directions of Adsorptive Therapies

Despite the promising benefits of adsorptive therapies, there are challenges that must be addressed. These include optimizing adsorbent materials, therapy duration, and managing potential side effects. Furthermore, more research is needed to fully understand the impact of these therapies on the quality of life of CKD patients [73].

## 6. Protein-Bound Uremic Toxin Displacers

The concept of displacing protein-bound uremic toxins (PBUTs) from their binding sites on plasma proteins, particularly albumin, offers a novel therapeutic approach in the management of chronic kidney disease (CKD). This strategy aims to increase the free fraction of these toxins, thereby facilitating their removal through dialysis.

### 6.1. The Role of Displacers

**Figure 2:**
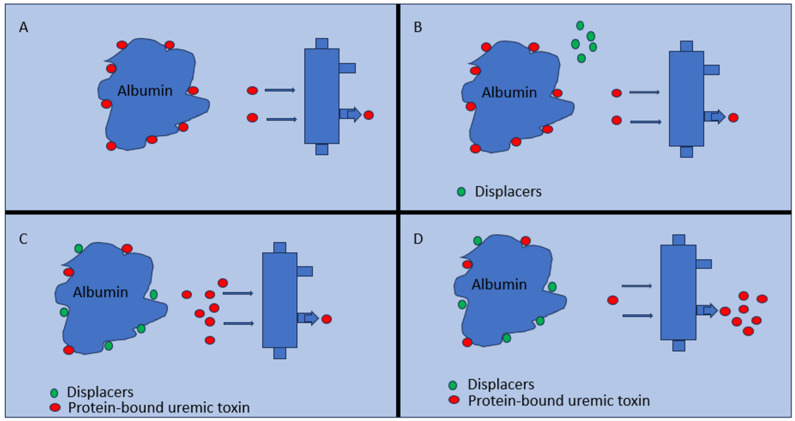
Mechanism of displacer action on albumin-bound uremic toxins. (**A**) Albumin (blue) with bound uremic toxins (red); (**B**) displacers (green) introduced; (**C**) displacers bind to albumin, releasing toxins; and (**D**) free uremic toxins increase post-displacement and are eliminated through hemodialysis.

Displacers work by competing with PBUTs for binding sites on plasma proteins. This competition results in an increased concentration of the free, unbound fraction of the toxins, which is more amenable to dialysis clearance. Albumin, the principal transporter protein in blood plasma, has specific subdomains that bind to toxins through non-covalent bonds. The competition for these binding sites by displacers is a critical mechanism for enhancing toxin removal [80]. Figure 2 show the mechanism of displacers’ action on PBUTs.

### 6.2. The Affinity for Albumin of Uremic Toxins (UTs)

Some uremic toxins, as mentioned, exhibit a notable affinity for proteins. These toxins predominantly bind to the Sudlow sites I and II on albumin [7,81]. This characteristic plays a crucial role in their pharmacokinetics and the difficulty of their removal through conventional dialysis procedures. Other uremic toxins, such as hippuric acid (HA), indole-3-acetic acid (IAA), and 3-carboxy-4-methyl-5-propyl-2-furanpropionate (CMPF), also show affinity to albumin, albeit to a lesser extent, as depicted in Table 1.

The affinity for albumin of the various studied molecules allows for an in vitro analysis of the competition occurring at protein binding sites when these compounds are administered as shown in Table 3. This competition leads to a decrease in protein binding sites and consequently an increase in the concentration of free UTs susceptible to dialysis [82].

### 6.3. Major Described Displacers

- Ibuprofen: A nonsteroidal anti-inflammatory drug (NSAID) with high protein-binding capacity, ibuprofen effectively displaces PBUTs such as p-cresyl sulfate (pCS) and indoxyl sulfate (IS) from albumin. However, its long-term use poses risks like gastrointestinal and renal complications [83]. Cellulose membranes embedded with ibuprofen have been developed, which exhibit a 1.2-fold increase in the removal of protein-bound uremic toxins (PBUTs). This performance is slightly lower than that achieved with ibuprofen perfusion, yet it comes without the associated potential risks [84].
- Furosemide: This diuretic shows a high affinity for albumin and can increase the free fraction of certain UTs like hippuric acid. Combined with ibuprofen, it enhances the displacement of toxins like IS [80].
- Tryptophan: Being the precursor of IS through intestinal microbiota metabolism, tryptophan shares structural similarities with some uremic toxins. It can bind to the Sudlow site II on albumin. A concentration of 1 mM of tryptophan increases the free fraction of IS and p-CS by a factor of 2, demonstrating its ability to compete with these toxins for albumin-binding sites [85].
- Non-Esterified Fatty Acids (NEFA): NEFAs have shown a high capacity to increase the free fraction of UTs such as IS and pCS [86]. However, high concentrations of these molecules are required to achieve this effect, which may predispose patients to adverse effects, and in the case of NEFA, there is a high risk of hemolysis at the concentrations necessary for the displacing effect on UTs.

**Table 3:** Effects of various displacers on the removal of PBUTs in dialysis therapies.

Displacer	Effect on PBUT Removal	Considerations	References
Ibuprofen (1 mM)	Free fraction of IS and pCS increased by a factor 3 No impact on HA removal	Handling high doses can be a risk for HD patients	[80]
Furosemide (1 mM)	Free fraction of IS and pCS increased by a factor of 1.3 HA by a factor of 1.5	Side effects such as ototoxicity	[80]
Ibuprofen + Furosemide	Increased the removal of IS by a factor of 3 and IAA by a factor of 2	Enhanced PBUT displacement but increased the risk of side effects	[80]
Tryptophan (1 mM)	Free fraction of IS and pCS increased by a factor of 2.0 No impact on HA removal	Could increase uremic syndrome	[80,85]
Non-esterified fatty acids (NEFAs)	High capacity to increase free fraction of IS and pCS	High doses required Risk of hemolysis	[86]
Salvianolic acids	In vitro, increased the dialysis efficiency of IS and pCS by 99.13% and 142.00%, and in vivo (rats), by 135.61% and 272.13%	Need to test these results in patients	[87]

- Salvianolic Acids: Salvianolic acids, including lithospermic acid (LA), salvianolic acid A (SaA), tanshinol (DSS), caffeic acid (CA), salvianolic acid B (SaB), protocatechuic aldehyde (PA), and rosmarinic acid (RA), are molecules with high affinity for albumin receptors, significantly increasing the free concentration of UTs. This effect depends on their plasma concentration [87].

### 6.4. Efficacy and Safety

While experimental studies have shown promising results with displacers, their clinical efficacy and safety are not fully established. Comprehensive clinical trials are required to validate their effectiveness in reducing UT levels over time [88].

The potential side effects and clinical limitations of long-term use of some displacers, such as ibuprofen and furosemide, necessitate careful consideration of their application.

### 6.5. Future Directions for Displacer Use

Some challenges in implementing these molecules as standard treatment include the following:

Risk of Side Effects: Certain displacers, such as ibuprofen or furosemide, have clinical limitations in chronic use, potentially causing unwanted side effects such as hypertension or decreased residual diuresis in the case of ibuprofen or furosemide-induced ototoxicity [89]. An additional concern is that displacement compounds may result in elevated intracellular concentrations of PBUTs, which are demonstrably more toxic than their bound counterparts.

Dosage and Administration: The complexity of treatment regimens in patients with chronic kidney disease is influenced by the types of molecules and doses studied so far. Ideally, displacers with minimal side effects and, if possible, even health benefits, such as fatty acids and tryptophan, should be chosen [82].

Need for More Research: More research is required to fully understand the dynamics of toxin binding and displacement under different physiological and pathological conditions, as well as to identify the most effective and safe displacers for clinical practice [82].

Cost-Effectiveness Considerations: The introduction of new therapies in clinical practice should also consider cost-effectiveness aspects, especially in the context of chronic kidney disease, where costs are already high.

## 7. Conclusions

The management of chronic kidney disease (CKD) and its complications, particularly relating to the accumulation of protein-bound uremic toxins (PBUTs), presents a complex and evolving challenge. This comprehensive review has explored the multifaceted aspects of PBUTs, from their definition, classification, and systemic effects, to the emerging strategies for their clearance and potential future treatments.

It is clear that PBUTs play a significant role in the pathology of CKD, contributing to a range of systemic effects, particularly on cardiovascular health and immune function. The binding of these toxins to plasma proteins, notably albumin, underscores the complexities involved in their clearance.

Conventional dialysis techniques, such as hemodialysis and peritoneal dialysis, have limited efficacy in removing PBUTs due to their high protein-binding nature. The length of dialysis sessions and the maintenance of residual renal function are crucial factors in enhancing toxin clearance. The exploration of online hemodiafiltration and the use of medium cutoff (MCO) dialyzers represent significant strides in improving dialysis efficacy. However, the effectiveness of these methods specifically for PBUTs needs further investigation and clinical validation.

Adsorptive therapies and the use of displacers offer promising avenues for more effective removal of PBUTs. Adsorptive therapies, particularly with activated carbon and other novel materials, show potential in enhancing toxin clearance. Displacers, such as ibuprofen, furosemide, and tryptophan, aim to increase the free fraction of PBUTs, thereby facilitating their removal. Yet, their clinical efficacy, safety, and long-term application require careful evaluation and further research. Critical areas for further exploration include the development and refinement of dialysis techniques and cleansing concepts tailored to the treatment of uremic patients, the investigation of a standard dialyzer with standard sorbent (activated charcoal) in dialysis with a high dialysate flow, novel adsorptive materials, and the clinical implementation of toxin displacers. Each of these areas presents its own set of challenges and opportunities, particularly in terms of efficacy, safety, and cost-effectiveness.

In conclusion, while significant progress has been made in understanding and managing PBUTs in CKD, ongoing research and innovation are critical. Future studies should focus on optimizing current treatment modalities, exploring new therapeutic strategies, and understanding the long-term implications of these treatments on patient outcomes and quality of life. The ultimate goal remains enhancing the standard of care for CKD patients, reducing the burden of uremic toxicity and improving overall health outcomes. At present, offering specific recommendations is challenging due to the complexities associated with PBUT elimination. The insights presented in this review are based on studies aimed at reducing PBUTs in clinical practice. Until more effective strategies are implemented, the most rational approach to eliminating these toxins in patients involves maintaining residual renal function. This necessitates proper and hypotension-free dialysis. Among various techniques, daily dialysis has been shown to achieve superior clearance. Time continues to be a critical factor in the effective removal of these molecules.

## Figures and Tables

**Table 1:** Albumin-binding percentages of common protein-bound uremic toxins (PBUTs) (adapted from Shi et al. [16]).

Uremic Toxin	Albumin Binding %
Indoxyl Sulfate (IS)	90–95%
p-Cresyl Sulfate (pCS)	90–95%
Hippuric Acid (HA)	>40%
Indole-3-Acetic (IAA)	>30%
3-Carboxy-4-Methyl-5-Propyl-2-Furanpropionate (CMPF)	>40%

**Table 2:** Efficacy of different clearance strategies for protein-bound uremic toxins (PBUTs), comparing dialysis techniques, the role of residual renal function, online hemodiafiltration, expanded hemodialysis, and adsorptive therapies. IS, indoxyl sulfate; p-CS, p-cresyl sulfate; HA, hippuric acid; IAA, indole acetic acid.

Clearance Strategy	Tested PBUT	Clearance Efficiency	Clinical Effects and Conclusions	Ref.
Dialysis Techniques				
Convectional HD	pCS and IS	Less than 50%	Alternative strategies promise to be more efficient	[48]
	pCS, IS and CMPF	29%, 32% and 0%, respectively		[49]
	pCS, IS and inorganic phosphate	No significant clearance	Need to focus on different approaches	[50]
Prolonged Convectional HD	pCS, IS, IAA, CMPF and HA	IAA, IS and pCS at the borderline of significance		[47]
	pCS	Significantly less than other soluble molecules	Convection can provide superior protein-bound solute removal compared with high-flux HD	[52]
Residual Renal Function	IS, pCS, IAA, HA, p-cresyl glucuronide, kynurenine, kynurenic acid	Only IS decreased by 8.0%	RRF is an important determinant of PBUT plasma concentrations in HD patients	[53]
	pCS and IS	1.7% and 2%, respectively	The implementation of theOWHD plus LPD strategy may be useful for lowering PBUTs	[54]
	IS, pCS, HA and phenylacetylglutamine	Significantly less than the rates of urea and creatinine	An increase in treatment frequency would be required to significantly reduce the plasma levels of PBUTs	[55]
Online HDF	pCS and IS	Free IS and free and total pCS remained unaltered	Current HDF techniques have only limited impact on IS and pCS plasma levels in the short and also long term	[56]
Expanded HD	IS and pCS	No statistically significant clearance	The clearance did not differ between the HF-HD, post-OL-HDF, and MCO-HD	[57]
	pCS and IS	No statistically significant clearance		[58]
Adsorptive Therapies				
Oral absorbents (AST-120)	IS	Dose dependent decreased levels	To determine whether this effect can attenuate the progression of CKD	[59]
	pCS, IS and phenyl sulfate	Reduction of IS (total 45.7%; free 70.4%) pCS (total 31.1%: free, 63.5%) and phenyl sulfate (free 50.6%)	AST-120 has additive effects on the continuous reduction of some PBUTs in anuric patients in HD	[60]
Activated charcoal	pCS and IS	Increase in the clearance of protein-bound solutes without altering the clearance of unbound solutes	Increasing the dialysate flow without the addition of sorbent, had a similar effect	[61]
Hexadecyl-immobilized cellulose bead (HICB)	IS, pCS, IAA and phenyl sulfate	34% decrease in free form, no change in total	Need to develop more effective materials to adsorb PBUTs selectively	[62]
Ordered nanoporous adsorbent material (CMK-3 type)	IS and HA	Significant reduction in the free form but not the total form	The IS removal is slightly lower than the corresponding one for HA	[63]
Divinylbenzene-polyvinylpyrrolidone (DVB-PVP)	IS and pCS	In vitro 54% IS and 56% PCS, In vivo efficient only for IS plasma levels	Symbiotic treatment with DVB-PVP HD decreased IS and pCS; this study provides the first line of evidence on the synergistic action of gut microbiota modulation and an absorption-based approach	[64]

## Data Availability

Not applicable.
